# Confinement‐Induced Ordering and Self‐Folding of Cellulose Nanofibrils

**DOI:** 10.1002/advs.201801540

**Published:** 2018-12-18

**Authors:** Kathleen Beth Smith, Jean‐Nicolas Tisserant, Salvatore Assenza, Mario Arcari, Gustav Nyström, Raffaele Mezzenga

**Affiliations:** ^1^ Department of Health Sciences and Technology Swiss Federal Institute of Technology in Zurich 8092 Zurich Switzerland; ^2^ Nanotechnology Group Swiss Federal Institute of Technology in Zurich 8803 Rüschlikon Switzerland; ^3^ Institute for High Frequency Technology Braunschweig University of Technology 38106 Braunschweig Germany; ^4^ Laboratory for Applied Wood Materials Empa 8600 Duebendorf Switzerland; ^5^ Department of Materials Swiss Federal Institute of Technology 8093 Zurich Switzerland

**Keywords:** alignment, cellulose nanofibrils, confinement, kink distribution, mesoscopic properties

## Abstract

Cellulose is a pervasive polymer, displaying hierarchical lengthscales and exceptional strength and stiffness. Cellulose's complex organization, however, also hinders the detailed understanding of the assembly, mesoscopic properties, and structure of individual cellulose building blocks. This study combines nanolithography with atomic force microscopy to unveil the properties and structure of single cellulose nanofibrils under weak geometrical confinement. By statistical analysis of the fibril morphology, it emerges that confinement induces both orientational ordering and self‐folding of the fibrils. Excluded volume simulations reveal that this effect does not arise from a fibril population bias applied by the confining slit, but rather that the fibril conformation itself changes under confinement, with self‐folding favoring fibril's free volume entropy. Moreover, a nonstochastics angular bending probability of the fibril kinks is measured, ruling out alternating amorphous–crystalline regions. These findings push forward the understanding of cellulose nanofibrils and may inspire the design of functional materials based on fibrous templates.

## 1 Introduction

Over the last few decades, materials science has taken a turn toward smart, green materials.^1^ One source of inspiration for such materials comes from nature itself, where the preprogrammed features of individual building blocks self‐assemble and fold into extremely complex and hierarchical structures.^2^, ^3^, ^4^ Although one most commonly thinks of DNA and proteins when considering such natural systems, another incredible natural material is cellulose,^5^ the most abundant organic polymer on the planet.^6^

Cellulose shows fascinating order and hierarchy over multiple lengthscales.^7^ This, combined with its high stiffness and specific strength, has made it a prime component in the fabrication of self‐assembled materials such as hydrogels,^8^ nematic liquid crystals,^9^ and even chiral nematic films.^10^ The assembly of cellulose into these highly ordered materials has been well studied,^11^, ^12^ as well as the structure of cellulose crystals on the atomic level.^13^ However, little is known about the structure and assembly of cellulose particles between these two length scales. Thus, the full potential of self‐assembled hierarchical cellulose materials is yet to be reached, as studying and understanding single particles at different length scales is paramount for rational material design.^14^ This concept is epitomized by DNA, where the assembly mechanisms of its different building blocks are so well established that one can engineer it to fold and self‐assemble into highly complex patterns.^15^, ^16^, ^17^ This has enabled the recent development of an impressive amount of DNA‐based applications and technologies.^17^, ^18^, ^19^, ^20^ In spite of its potential, precisely self‐assembled cellulose nanostructures and materials are still to be realized, due to the lack of detailed understanding of the mechanisms ruling mesoscopic structures.

One of the smallest building blocks used in cellulose‐based self‐assembled materials is cellulose nanofibril (CNF).^21^ It is known that the processing of the cellulose can influence the length, aspect‐ratio, and degree of polymerization within the fibrils.^22^, ^23^ It has also been discussed that the kinks along the fibrils may be due to mechanical treatment and hence depending directly on the processing conditions. Furthermore, the cryo‐scanning electron microscopy (SEM) and atomic force microscopy (AFM) images reported in this study lead to the conclusion that kinks are also present to a certain extent in solution.^24^ Theoretical work has been done by Ciesielski et al.^25^ where the kinks were described by energy minimization along certain crystalline planes. However, there is still no clear explanation as to their exact nature.

In order to better understand the structural properties of the CNFs, how to manipulate the mesoscopic features, and gain further insight on the nature of the kinks in view of its relevance for practical applications, the effect that confinement has on the individual fibrils was studied. Confinement was chosen as it has been extensively studied both theoretically^26^, ^27^, ^28^ and, in recent years, experimentally.^29^, ^30^, ^31^, ^32^, ^33^ It is known to reveal structural information or change in topology of the constrained elements.^29^, ^32^, ^34^ A noteworthy example of this was observed by Japaridze et al.^32^ in the case of circular DNA, where they observed hairpins at the nicked regions of the DNA plasmids when under strong confinement. These structural defects could be analogous to the kinks observed in CNFs, bringing a strong motivation to study them under confinement. In addition, confinement is known to create order and alignment, a crucial parameter that bioinspired materials try to reproduce.^3^, ^35^ Therefore, confinement can provide more than just much‐needed structural information, it can also give an overall picture of how the fibrils and other similar biomolecules behave during certain material processing where confinement by geometry and increase in concentration are used to induce macroscopic ordering and self‐assembly.^35^, ^36^, ^37^

Most confinement experiments are accomplished using microfluidics combined with optical imaging and staining of the molecules.^29^, ^30^, ^31^ In this study, our goal was to obtain high‐resolution images while avoiding perturbations of the fibril structure due to staining. This was achieved by utilizing a novel thermal scanning probe lithography technique^38^ to create confinement patterns, in which fibrils were deposited by adsorption. To avoid additional effects due to fibril–fibril interactions, the concentration of deposited CNFs was tuned, so that the area available to each fibril within the slits was larger than the 2D excluded volume derived from Onsager's principle.^39^ The CNFs were then imaged under confinement using AFM techniques, and subsequently single molecule statistics was performed on the confined fibrils. Our results show the expected alignment and most surprisingly, a net folding of the CNFs. In order to determine whether the slits were acting as topological filters for fibrils with a higher kink count, geometry‐based simulations were carried out. Simulations consisted in testing which fibrils, generated from the unconfined statistics, would fit into an implemented confined geometry. The subsequent subpopulation showed that the observed net‐folding could not be explained by selectivity of the slits for a specific fibril population, but rather by a conformational change of the fibrils themselves. Further a preferential bending direction of the CNFs was observed, which provides additional experimental evidence to the fact that amorphous and crystalline regions cannot alternate along the contour length of CNFs.

## 2 Results

**Figure 1:**
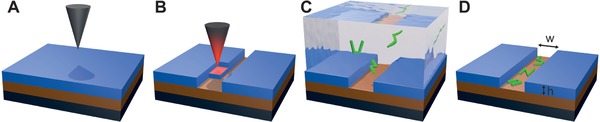
Experiment overview. A) Stack of prepatterned substrate. Layers from bottom to top: silicon wafer, P4VP, and PPA. B) Patterning of the substrate by thermal probe lithography. C) Deposition of the CNFs by adsorption through specific electrostatic interactions. D) Final sample displaying folded CNFs in a slit of width *w* and height *h*.

The confinement of cellulose nanofibrils of average contour length 〈*L*〉 ≈ 600 nm and average height 〈*h*〉 ≈ 1.5 nm (see Figure S1 in the Supporting Information for height distribution) was studied on prepatterned poly(phthalaldehyde) (PPA)/poly(4‐vinylpyridine) (P4VP) substrates (see the Experimental Section) having rectangular slits of depth *h* = 60 nm and width *w* varying between 0.75 and 9.50 µm, **Figure** **1**. PPA is a thermally cleavable polymer that can be removed locally by a thermal probe as shown in Figure 1A,B. P4VP is a polycation chosen to attract the fibrils by electrostatic interaction. The degree of confinement in such patterns can be quantified by 〈*L*〉/*w*. As depicted in panels (C) and (D) of Figure 1, CNFs display sharp bends in otherwise stiff segments. These bends are referred to as kinks. The main traits of the CNFs are the number of kinks, the kink angles, and the contour length. All further characterizations of the fibrils are constructs derived from these features. In addition, the area occupied by each fibril can be characterized by the normalized mean square radius of gyration ${\left(R_{g}/L\right)}^{2}=\frac{1}{NL^{2}}\sum_{i}^{N}\left\langle {\left({\vec{r}}_{i}-{\vec{r}}_{\mathrm{cm}}\right)}^{2}\right\rangle$, with ${\vec{r}}_{i}$ the segment vector *i* along the contour length, and ${\vec{r}}_{\mathrm{cm}}$ the center of mass of the chain.

### 2.1 Alignment

Confinement is well known to induce an orientation of the object along the long axis of the slit. In the case of a rigid rod, the degree of alignment in a slit has an exact analytical solution. For ideal flexible and semiflexible chains, seminal theoretical work has been done by de Gennes^26^ and Odjik,^27^, ^28^ where de Gennes elaborates his “blob” theory under weak confinement, e.g., *R*_g_ ∼ *w*, with *R*_g_ the radius of gyration, and Odjik describes the chain as a series of deflecting segments when under strong confinement, e.g., *R*_g_ ≫ *w*. The kinks in CNF make it an interesting case somewhere between a rigid rod and semiflexible polymer. This justifies the use of 〈*L*〉/*w* to quantify the degree of confinement.

**Figure 2:**
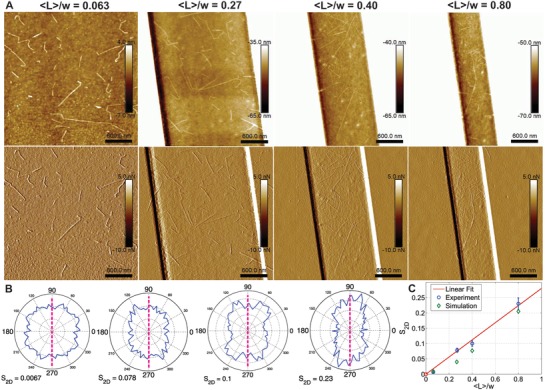
Alignment of cellulose in slits. A) AFM images (top height, bottom peak force error channel) of cellulose in slits of 〈*L*〉/*w* ratios of 0.063 (unconfined), 0.27, 0.40, and 0.80 (left to right). B) Full statistical orientation distributions of fibrils in slits for 〈*L*〉/*w* ratios of 0.063, 0.27, 0.40, and 0.80 (left to right), where the dashed pink line represents the slit long‐axes. C) Experimental *S*_2D_ (blue circles) and simulated *S*_2D_ (green diamonds) as a function of 〈*L*〉/*w*. Linear fit (red line) forced through 0, of *R* ^2^ of 0.988 and RMSE of 0.0101.

In **Figure** **2**A AFM images were chosen to show this effect on the CNFs in slits of 〈*L*〉/*w* ratios of 0.063, 0.27, 0.40, and 0.80. This alignment was quantified in two ways: by plotting the orientation distribution (OD), as shown in Figure 2B, and by calculating the *S*_2D_ order parameter given by *S*_2D_ = 〈cos (2θ)〉,^40^ where θ is the angle between the *n*th segment and a local director.

The OD plots clearly show a trend from isotropic to anisotropic average particle direction. It can be observed that though the component in direction of the confinement gets increasingly narrower, the rectangular shape is well preserved. This is attributed to the distinct morphology of the nanocellulose fibrils.

The *S*_2D_ order parameter increases linearly as a function of confinement, as seen in the Figure 2C. (This fit has a *R* ^2^ of 0.988 and RMSE of 0.0101). It was assumed in the zero intercept of this fit that an infinite slit width would yield a purely isotropic fibril distribution. To better understand this trend, the values were compared to those of simulated CNF in confinement (green diamonds). A full explanation of the simulations can be found in the Experimental Section. In short, fibrils of fixed contour length 〈*L*〉, with corresponding statistics extracted from the distributions of the unconstrained case, were generated and placed in a slit of width *w*. If the fibril fit into the slit it was taken into the statistics, if not it was discarded. The simulated values of *S*_2D_ were obtained by simulating fibrils in slits with various *L*/*w* ratios and taking the sum of these *S*_2D_ weighted according to the experimental contour length distributions normalized by *w*. We observe that the simulated values are systematically lower for the confined cases. This can be justified by the fact that we used a fixed kink angle, that the implemented statistics are exact only for 〈*L*〉, and that the simulations do not take the fibrils excluded volume into account.^26^, ^33^ The excluded volume was therefore estimated from that of a rigid rod in 2D (for this reason we will refer to an excluded area). For a CNF of same length, this approximation should overestimate the actual excluded area. From Onsager's theory,^39^ we calculated the excluded area for rigid rod in the limit of *L* ≫ *d*, with *d* the diameter of the rod, to be $\frac{2L^{2}}{\pi }$ (see Supporting Information). If we compare this to the area available to each fibril (values extracted from AFM images), see Figure S2 in the Supporting Information, the estimated values showed that in certain cases, especially at the ratio 〈*L*〉/*w* = 0.27, we could have an additional alignment from the fibrils interacting with each other.

### 2.2 Fibril Conformation

**Figure 3:**
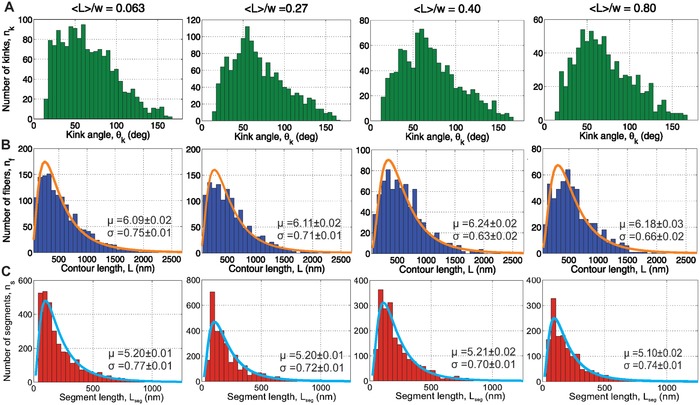
Full statistics distributions. In all distributions the number of counted fibrils for the different confinements are: 0.063–1420, 0.27–1286, 0.40–791, and 0.80–562. A) Kink angle distributions; B) fibril contour length distributions with lognormal fit (μ and σ fit parameters given on the plot). C) Segment length distributions with lognormal fit (μ and σ fit parameters given on the plot).

It has been reported that confinement can influence the conformation of the confined elements.^29^, ^32^, ^34^, ^41^ Consequently, different fibril attributes were investigated, the full detailed distributions of which can be seen in **Figure** **3**. We observe a shift toward higher values in the kink angle distribution under confinement, as well as a decrease in the tail of the segment length distributions. The contour length, however, is left more or less undisturbed. Let us note that we can attribute any change in conformation to the effect of confinement, as we processed all substrates identically to make up for any bias due to absorption (see the Experimental Section).

**Figure 4:**
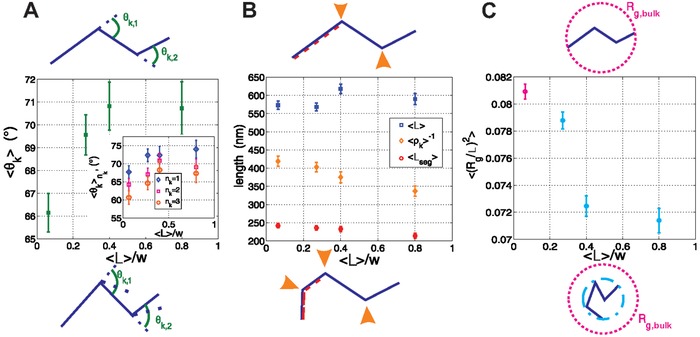
Fibril conformations in slits. Schematics at the top represent fibril parameters for the unconfined case, whereas those at the bottom represent the same parameters in the confined cases. A) Overall mean kink angle, 〈θ_k_〉, as a function of 〈*L*〉/*w*, with subplot of the mean kink angle per kink population, ${〈\theta_{k}〉}_{n_{k}}$ as a function of 〈*L*〉/*w*. B) Mean contour length 〈*L*〉 (blue squares), mean inverse kink density 〈ρ_k_〉^−1^ (green diamonds), and mean segment length 〈*L*_seg_〉 (red circles) as a function of 〈*L*〉/*w*. C) normalized mean square radius of gyration 〈(*R*_g_/*L*)^2^〉 as a function of 〈*L*〉/*w*.

To better visualize the effect of the slits on CNFs, mean values of fibril characteristics were extracted from the full statistics and plotted as a function of confinement, **Figure** **4**. For each parameter, corresponding schematics depict the fibril conformation in the unconfined and confined states. Figure 4A shows the mean kink angle 〈θ_k_〉 as a function of confinement, with a subplot of ${〈\theta_{k}〉}_{n_{k}}$ for fibril subpopulations, identified by different number of kinks, *n*_k_ = 1, 2, 3. We observe that the overall 〈θ_k_〉 increases in confinement. We also note that the ${〈\theta_{k}〉}_{n_{k}}$ follows a similar trend with confinement, but also that ${〈\theta_{k}〉}_{n_{k}}$ monotonically decreases with *n*_k_.

Figure 4B shows the mean contour length 〈*L*〉, the mean inverse kink density 〈ρ_k_〉^−1^ = 〈(*n*_k_/*L*)〉^−1^ (where *n*_k_ is the kink number), and the mean segment length 〈*L*_seg_〉 = 〈*L*/(*n*_k_ + 1)〉. Nyström et al.^42^ exploited these parameters to understand if the fibrils break along a segment or at a kink, or if a new kink is observed. Using their classification system, we could determine that we are observing more kinks per fibril in confinement (as our 〈*L*〉 is constant in the different slits but the 〈ρ_k_〉^−1^ and the 〈*L*_seg_〉 decrease).

The combination of these two panels helps to understand the results of Panel 4C. An overall decrease in the normalized 2D mean square radius of gyration 〈(*R*_g_/*L*)^2^〉 is apparent once the fibril is confined. Taking into account that we observe both fibrils with more kinks and with higher kink angles, this result is expected as on average the fibrils are folding upon themselves under increasing confinement.

These results can be understood from an entropic point of view, as fibrils that are more bent can adopt more states in the slit than stretched out fibrils.^34^ In other words, while the change in configurational entropy of the fibrils is essentially equivalent between stretched and folded states, folding increases free volume entropy of the fibrils in the slit. It should be noted that this observation holds in a regime of weak confinement as 〈*L*〉 ∼ *w*, and that this effect cannot be extrapolated to strong confinement, which goes beyond the limits of our experimental procedure.

To determine if these results could be justified solely from a slit selectivity for existing fibril populations that would be entropically favorable, geometry‐based simulations of the cellulose fibrils under confinement were performed (see the Experimental Section for details). The simulations showed a similar trend for the mean kink number 〈*n*_k_〉, with an increase from 1.24 to 1.29 (i.e., 4%). However, the shift observed in the experiments was from 1.24 to 1.69 (37%), see Figure S3 in the Supporting Information. It could be argued that the averaged values used in the simulation are the source of this discrepancy. However, this possibility was discarded by performing a second set of simulations with CNFs of constant segment lengths and a maximum kink angle up to 160°. These features are typical of the fibrils occupying the smallest area observed in experiments, thus considering such a subpopulation provides an upper‐boundary to the prediction range of a purely selective model. This second set of simulations showed only an increase in 〈*n*_k_〉 of 10%, i.e., it still did not account for the experimental data. Therefore, it was determined that the geometry of the system is not solely acting as a conformational filter, but that it also has a direct influence on the conformation adopted by the nanofibrils. A model capable of quantitatively recapitulating the data would thus need to account for more complex features, such as detailed mechanical and structural features of single fibrils, adsorption dynamics, etc., but is well beyond the scope of this paper.

### 2.3 Kink Populations

Usov et al.^24^ reported that the kink angle distribution of the nanocellulose does not follow a Gaussian distribution and is nonsymmetric. This argument was used to support one of the two models^43^ used to describe the disposition of the ordered and disordered regions in cellulose fibrils and to suggest that the kinks were possibly a result of the sonication used in the preparation of the nanocellulose. Their results are in favor of the model of a crystalline core and amorphous shell. This evidence went also in the direction of the results of other studies.^8^, ^44^, ^45^, ^46^, ^47^ In this section, a similar argument to that of Usov et al. is proposed to further push the evidence towards the aforementioned model.

**Figure 5:**
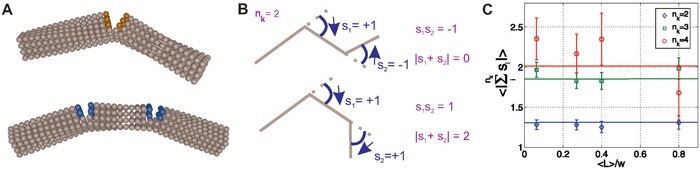
Kink bending. A) Depiction of a fibril with one kink (top) breaking and bending more than a fibril with two kinks (bottom). Colored beads represent the broken bonds. B) Schematic of the kink bending direction *s*_k_, for a kink population *n*_k_ = 2. Top: fibril with kinks bending in opposite directions, *s*_1_ = −*s*_2_. Bottom: a fibril with kinks bending in the same direction *s*_1_ = *s*_2_. C) $〈|\sum_{i=1}^{n_{k}}s_{i}|〉$, for kink populations *n*_k_ = 2, 3, 4. Solid horizontal lines represent the theoretical values using a probability *p* = 0.65 of kinks bending in the same direction as the previous kink.

First, as observed earlier, the mean kink angle ${〈\theta_{k}〉}_{n_{k}}$ decreases systematically with increasing *n*_k_ (Figure 4A). We propose to interpret these results as follows. Let us suppose kinks are formed from breaking to release an inflicted strain. If the rupture happens in only one place, all the energy goes into breaking the fibril in that one spot, resulting in one deep break, see **Figure** **5**A (top). Conversely, if two spots are involved the energy is shared between the two areas and therefore the breakage will not go as deep, see Figure 5A (bottom).

A further argument in favor of a stress‐induced origin of kink formation comes from an analysis of kink bending directions. Let us look at the populations of fibrils with two or more kinks, and assign a sign to the direction in which the fibril bent, see Figure 5B. For instance, following the contour of the fibril, if the fibril bent to the right we determined the sign *s*_k_ = +1, if it bent to the left *s*_k_ = −1. Using these *s*_k_ values we calculated 〈*s*_1_ *s*_2_〉 for the population of fibrils with two kinks. Assuming the probability of bending in either direction were that of a random walk, a mean value of 0 and a variance of 1 would be expected. However, a mean of 0.30 and variance of 0.91 for unconfined fibrils were calculated. From this, the probability *p* of bending in the same direction is obtained by 〈*s*_1_ *s*_2_〉 = *p* − (1 − *p*), giving *p* = 0.65 ± 0.03. The fibrils are therefore bending in a preferential direction, which is not in agreement with the alternating amorphous–crystalline regions model,^48^ and which indeed leads to a nonsymmetric, nongaussian distribution of the kink angle distribution (see Figure 3A). This finding therefore further supports the hypothesis that kinks are a product of mechanical strain. Indeed it is significantly more probable that they break in the same direction, if they are created by the same mechanical event (see Figure S4 in the Supporting Information). Accordingly a log‐normal segment length distribution is found in between kinks (see Figure 3C).

In addition, we calculated, $〈|\sum_{i=1}^{n_{k}}s_{i}|〉$, for *n*_k_ = 2, 3, 4, both from the data and from the probability *p* to bend in the same direction as given by Equations (1)–(3). The arbitrarity in the sign assignment, which is introduced in attributing right or left from a given fibril end taken as origin, is removed by taking absolute values of the averages (see Figure 5B). In general, a good agreement between this simple theory and the experimental values was observed, Figure 5C.

The constant sum values (as well as constant kinks per fibril within the slit, see Figure S5 in the Supporting Information) support the following hypothesis; that the aforementioned increase in kink number under confinement, is due mainly to pre‐existing unobservable kinks becoming apparent when the fibrils adopt a higher degree of folding to gain in entropy. Indeed, Usov et al.^24^ reported that it is hard to distinguish kinks below an angle of 20°. In addition, if new kinks were being introduced through confinement we would expect a decrease in the values of the sum as a function of confinement, as the bending of the fibril would be independent of the direction of the pre‐existing kinks. Only the case of four kinks shows discrepancies with the supposed model. It is hard to ascertain if this is from poor statistics or maybe an effect of the confinement. Therefore, from this data, we cannot exclude the possibility that extra kinks might be formed due to the confinement itself.

## 3 Discussion and Conclusions

In this study, the effect of weak confinement on individual CNFs has been explored. Alignment was observed and quantified. In addition, we observed an accentuation of the folding of the individual fibrils under confinement. Simulations showed that this effect could not be solely explained by a specific selectivity of the slits for pre‐existing fibril populations, but rather confinement itself induces the conformational change. To our knowledge this is the first time the folding of cellulose upon itself under confinement has been reported. In most cases, the high concentration of CNFs prevent a detailed characterization of the single particles, however this was overcome in our approach, by creating the confinement solely through geometry. This effect should be considered when developing materials using cellulose nanofibrils, as often confinement, be it by microfluidics, geometry, or high‐concentration, is used to encourage self‐assembly into higher ordered materials.^35^, ^36^, ^49^

As a practical example, our results may have interesting implications for nematic films,^11^ cholesteric droplets,^37^ and tactoids.^42^ As the kinks impede self‐assembly, these systems need to be generated by short rod‐like particles. If on top of this the fibrils are further folding upon themselves when at a concentration equivalent to weak confinement, this effect could shift up the expected critical concentration or even fully prevent the self‐assembly of the fibrils into such phases.

The accentuated kink angle under confinement as well as the fairly constant $〈|\sum_{i=1}^{n_{k}}s_{i}|〉$ for the different kink populations, lead us to suggest that a weak confinement experiment offers more information on the fibril characteristics, by bringing into observation kinks or weak regions that were not noticeable in the unconfined state, much like in the case of the circular DNA studied by Japaridze et al.^32^ In addition, a monotonic decrease in average kink angle as a function the increasing number of kinks, ${〈\theta_{k}〉}_{n_{k}}$ was observed. This trend and that of the preferential bending direction, lead us to suggest mechanical breaking of the fibrils due to the CNFs processing. This evidence further rules out the alternating amorphous–crystalline model for kink formation.^48^

In conclusion, geometrical confinement of cellulose nanofibrils enhances characterization possibilities, thus bringing us one step closer to understanding these nanomaterials at the lengthscale of the single particles. By comparing the statistical analysis of nanofibrils conformation based on experimental AFM images with simulations on entropy driven selectivity of the confining slit, it was possible to conclude that the confining geometry not only leads to orientational ordering of the fibrils, but it also has an influence on the conformation of the nanofibrils themselves. The most remarkable feature of this morphology‐controlled confinement is the self‐folding on the cellulose nanofibrils in order to increase the overall free volume entropy in the slit. The understanding of the implications of confinement of cellulose nanofibrils in simple geometries may guide and inspire the design of those functional nanomaterials where CNFs have emerged as main building blocks, with far reaching consequences in soft condensed matter, nanotechnology, and materials science.

## 4 Experimental Section

*Substrate Preparation*: 1 cm^2^ silicon wafer (P/Bor 〈100〉, thickness 525 µm, 1–30 ohm cm) pieces were cleaned by sonication in toluene, isopropanol, and milli‐Q water for 20 min each. The wafer surface was activated for 30 s in an O_2_ plasma cleaner.

A solution (10 mg mL^−1^) of P4VP *M*_w_ 60 000 Da (Sigma Aldrich) in 1‐butanol and a solution (5 mg mL^−1^) of linear PPA (Sigma Aldrich lot # is MKCC5806) in anisole were prepared for spin coating. The P4VP was chosen for its polyelectrolyte properties, at the pH of the cellulose sample it is positively charged, therefore the opposite charge of the nanocellulose fibrils. The PPA acts as a resist in the nanolithography technique used.

A 20 nm P4VP layer was spin‐coated onto the freshly activated wafer (100 µL at 4000 rpm for 1 min). 60 nm of PPA was spin‐coated (100 µL at 4000 rpm for 1 min) on top of the P4VP layer. The substrates were then baked for 3 min at 90 °C, to evaporate any extra solvents. The 20 nm of P4VP and 60 nm PPA were chosen to maximize both the writing of the patterns and the cellulose deposition. The initial layer of the polyelectrolyte is crucial for the adsorption of the cellulose, but also allows for a deeper patterning of the resist (as it acts as an insulating layer to the wafer that can act as a heat sink). The 60 nm of resist was needed to screen the full effect of the polyelectrolyte to the cellulose.

*Nanolithography*: The principle of the nanolithography technique used is fully described elsewhere^38^ and was carried out on a Nanofrazor apparatus (Swisslitho). In a few words, an AFM tip was heated to a temperature at which the PPA resist starts to evaporate, due to self‐amplified depolymerization. This reaction decomposes the polymer into its volatile monomer. The coupling of this effect with the AFM feedback allows for a high precision nanolithography. In the case of this study, simple geometries were chosen to study the confinement. Rectangles with widths of 9.5, 2.25, 1.5, and 0.75 µm, constant lengths of 18.5 µm, and constant depth of 60 nm. The rectangle of 9.5 µm was used as a control of the unconfined state. This control was favored over a plain P4VP layer because the lithography treatment could leave residues or change some properties of the layer due to the extreme heating, and the P4VP and the PPA could be mixed at some point of the process. Having the same substrate properties in the control is crucial, as any bias on fibril conformation due to the adsorption process or sample preparation is therefore the same in every experiment.

The parameters of the AFM were chosen to obtain the desired depth. The selection of these parameters varied from tip to tip. In general, a high temperature of 900 °C and a blunted tip were needed to reach a depth of 60 nm. At this depth one tip could write up to ten patterns before contamination took over. On average five good patterns were written per wafer.

*Carboxylated Cellulose Nanofibrils*: Carboxylated cellulose nanofibrils with a charge density of 0.65 mmol g^−1^ cellulose were prepared from never‐dried sulfite softwood‐dissolving pulp (Domsjö, Sweden) using a protocol for TEMPO‐mediated oxidation with 5 mmol NaClO per gram cellulose.^21^ Using ultrasonication, 100 mL of the cellulose pulp dispersion (0.5 g L^−1^) was fibrillated with a sonication probe (7 mm, 20% amplitude, 200 W, no interval) for 7 min. To remove nondissolved aggregates, the dispersion was centrifuged at 4000 rcf for 60 min.

*Fibril Deposition and AFM*: The bulk solution of cellulose fibrils was diluted 1000 times for the AFM sample preparation. A 100 µL drop of this solution was deposited onto the patterned substrates. After 3 min, the drop was gently rinsed off and the sample was gently dried by air stream. This protocol was chosen as it leads to a concentration of adsorbed fibrils lower than the critical concentration at which fibril–fibril interaction would be expected (see the “Excluded area” in the Supporting Information). AFM images of the patterns were taken using a Bruker FastScan in the scan assist mode or a MultiMode 8 in soft tapping mode. The cantilevers in both cases were RTESPA‐150 with a resonance frequency *f*_0_ of 150 kHz and a pring constant k, of 6 N m^−1^. Scan size was 25 × 25 µm and a minimum pixel resolution of 3072 pixels per line. Images were saved in the height channel and the peak force error channel.

*Image Analysis*: Single molecule statistics were carried out using an in‐house Matlab‐based software, FiberApp,^40^ to trace the individual cellulose fibrils. The features of the software are fully described elsewhere^40^ and additional information on cellulose tracing with this software can be found in Usov et al.^24^ The built‐in functions of the software provided the fibril contour lengths, kink angles, and individual orientation distributions. From these and the coordinates of the fibrils and kinks, additional information was extracted in Matlab, such as the fibril segment length, kink density, kink angle orientation, mean radius of gyration, the summed orientation distributions over all the images (with error bars performed by bootstrapping), positions of kinks, and fibrils in slits.

From the kink angle orientation, the sum of the kink angle signs could be theoretically calculated by assuming a probability *p* of bending in the same direction as the previous kink (assuming only nearest‐neighbor interactions), $\left\langle \;\left|\sum_{i=1}^{n_{k}}s_{i}\right|\right\rangle$ for different kink populations as a function of *p*. Considering all the possible conformations (see Figure S6 in the Supporting Information) we get the following expressions

(1) $$n_{k}=2,\;\left\langle \;\left|\sum_{i=1}^{2}s_{i}\right|\right\rangle =2p$$

(2) $$n_{k}=3,\;\left\langle \;\left[\sum_{i=1}^{3}s_{i}\right]\right\rangle =2p^{2}+1$$

(3) $$n_{k}=4,\;\left\langle \;\left|\sum_{i=1}^{4}s_{i}\right|\right\rangle =4p^{3}-4p^{2}+4p$$

In our confinement experiments, only the CNFs fully in the slits were taken into our statistics. To check if a fibril was in the slit, the height image was systematically compared with the equivalent peak force error image, as seen in Figure 2A. The AFM images analyzed in FiberApp were pre‐processed with a 3rd order flattening provided in the NanoScope Analysis software.

*Simulations*: Geometry‐based simulations were carried out by implementing the statistics of the unconfined CNFs in a C++ program and artificially confining them.

Specifically, the cellulose fibrils were implemented with a fixed contour length, fixed kink angles, and random segment lengths. In this case, fibrils between 550 and 650 nm were chosen, yielding a population of 104 fibrils with the following kink population probabilities $p_{n_{k}}$, **Table** **1**.

**Table 1:** Probabilities of having *n*_k_ kinks from the unconfined statistics of fibrils of lengths between 550 and 650 nm

*n*_k_	0	1	2	3	4
$p_{n_{k}}$	0.21	0.45	0.26	0.05	0.03

The preferential bending direction of the kink populations was set to the calculated one of *p* = 0.65. A fixed mean kink angle 〈θ_k_〉_0.063_ = 65° for the unconfined geometries was utilized, and 〈θ_k_〉_0.80_ = 70° for the most confined case.

From these statistics, multiple fibrils were randomly generated, with random overall orientation and random positioning of the center of mass in the slit. If the whole fibril fit into the implemented slit, it was accepted into the statistics, if not discarded. The same statistics as in the image analysis could then be carried out. In the case of fibrils with no kinks, the program was cross‐checked with the analytical solutions of rigid‐rods.

## Conflict of Interest

The authors declare no conflict of interest.

## Supporting information

Supplementary
