# Supplementary material for: Confinement‐Induced Ordering and Self‐Folding of Cellulose Nanofibrils
Source: Adv Sci (Weinh). 2018 Dec 18;6(4):1801540. doi: 10.1002/advs.201801540 (PMC6382315; doi:10.1002/advs.201801540)
Supplement: Supplementary file 1 — Supplementary [file ADVS-6-1801540-s001.pdf]

Copyright WILEY-VCH Verlag GmbH & Co. KGaA, 69469 Weinheim, Germany, 2018.

Supporting Information

for *Adv. Sci.,* DOI: 10.1002/advs.201801540

Confinement-Induced Ordering and Self-Folding of Cellulose Nanofibrils

*Kathleen Beth Smith, Jean-Nicolas Tisserant, Salvatore Assenza, Mario Arcari, Gustav Nyström, and Raffaele Mezzenga**

Supporting information: Confinement-induced ordering and self-folding of cellulose nanofibrils

Kathleen Beth Smith, Jean-Nicolas Tisserant, Salvatore Assenza, Mario Arcari, Gustav Nystrm, and Raffaele Mezzenga*

E-mail: raffaele.mezzenga@hest.ethz.ch

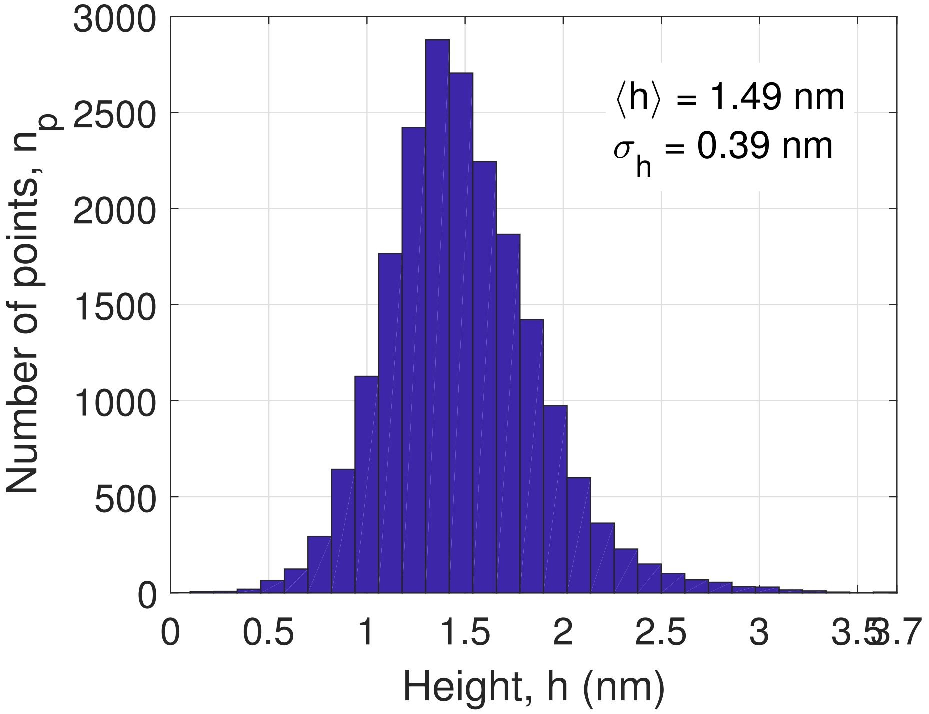

Figure 1: **CNFs height distribution** Typical height distribution of CNFs deposited on mica.

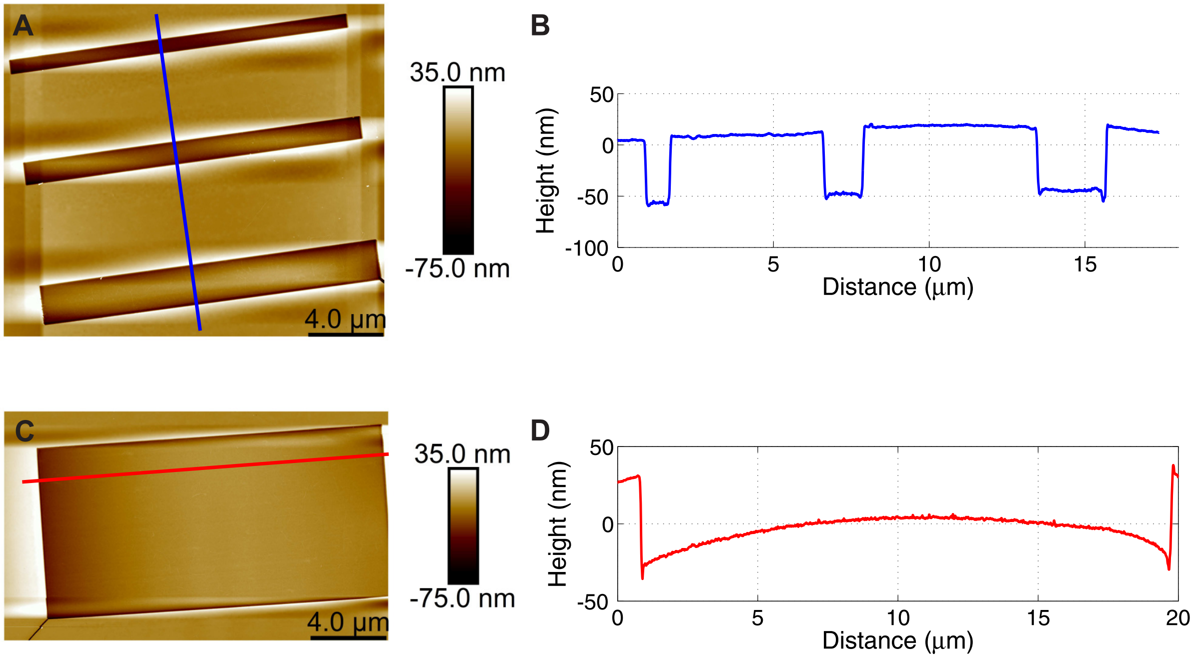

Figure 2: **Confinement patterns as in main article A** AFM image of the patterns of widths $w = 0.75,\ 1.50,\ 2.25$ from top to bottom. **B** Height profile along the blue line in the AFM image in **A**, the depth of the patterns are of 60 nm. **C** AFM image of the pattern of width $w = 9.5$ used for the unconfined case. **D** Height profile along the red line in the AFM image in **C**, the depth of the pattern is 60 nm.

Excluded area

Using Onsager's principle for calculating the excluded area of a rigid rod of length L and diameter d in 2D and in the limit where $L >> d$, we get:

$$A_{ex} = \frac{2}{\pi}\int_0^{\frac{\pi}{2}} L^2 \sin\theta d\theta = \frac{2L^2}{\pi}$$

We use this to estimate the excluded area for cellulose nanofibrils, where we take $< L >$ for L, with $A_{ex,\ 0.80} \approx 0.22\ \mu m^2$, $A_{ex,\ 0.40} \approx 0.24\ \mu m^2$, $A_{ex,\ 0.27} \approx 0.21\ \mu m^2$, and $A_{ex,\ 0.063} \approx 0.21\ \mu m^2$. The approximation of the available area per fibrils was extracted from the AFM images by calculating the area of the slit divided by the number of fibrils in each slit,

$A_{av} = A_{slit}/n_{fib}$. The following lists indicate the values in μm^2 extracted for the different slits used in the statistics. (In red we highlight the areas, that are lower than A_{ex}, in orange those that are slightly higher than A_{ex}).

- $\mathbf{A_{av,\ 0.80}}$: 1.98, 0.51, 0.77, 3.47, 6.94, 0.99, 0.99, 1.26, 1.39, 1.73, 1.98, 2.31, 0.93, 0.43, 0.29, 0.36, 1.98, 0.77, 2.31, 0.51, 0.66, 0.73, 3.47, 3.47, 0.53, 0.56, 0.73, 0.43, 0.73, 6.94, 0.66, 0.28

- $\mathbf{A_{av,\ 0.40}}$: 3.96, 0.43, 0.96, 2.52, 3.47, 3.96, 1.11, 1.16, 1.03, 0.87, 0.96, 0.68, 1.98, 1.21, 0.99, 1.03, 1.39, 0.36, 3.08, 1.63, 1.98, 1.73, 1.46, 1.39, 1.98, 1.54, 0.65, 0.57, 1.11, 9.25, 1.32, 6.94, 1.11

- $\mathbf{A_{av,\ 0.27}}$: 0.24, 0.21, 0.51, 0.20, 0.50, 0.44, 0.95, 0.89, 1.89, 1.89, 0.63, 2.19, 3.78, 1.73, 0.95, 2.19, 1.02, 2.78, 2.60, 1.67, 1.10

- $\mathbf{A_{av,\ 0.063}}$: 0.40, 2.31, 1.63, 1.46, 1.67, 2.55, 0.61, 3.45, 1.11

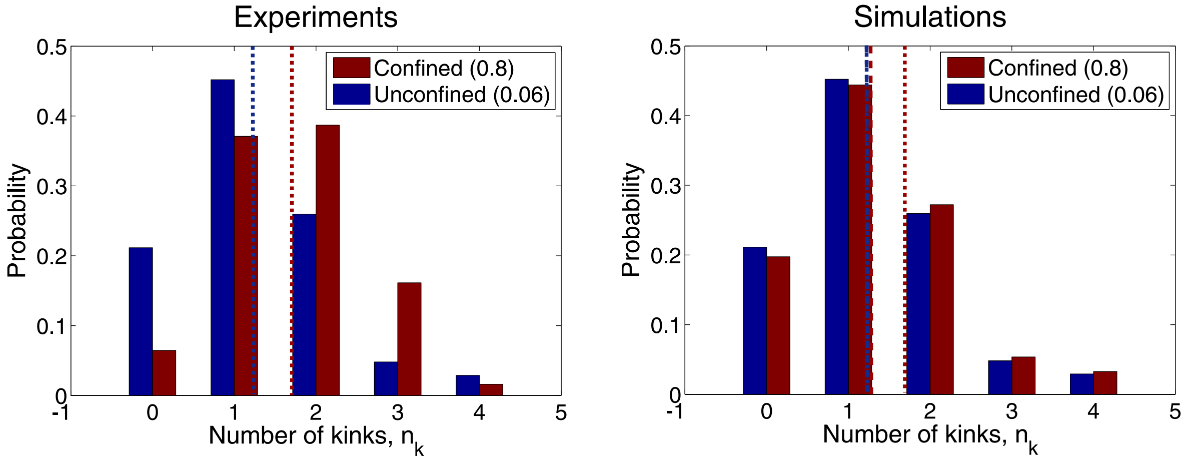

Figure 3: **Experiments versus Simulations.** (left) Experimental distribution of kink number in the unconfined $< L > /w = 0.06$ (blue) and confined $< L > /w = 0.8$ (red) cases. Dotted lines represent the mean values of the distributions 1.24 unconfined (blue) and 1.69 confined (red). (Right) Simulated distribution of kink number in the unconfined $< L > /w = 0.06$ (blue) and confined $< L > /w = 0.8$ (red) cases. Dashed lines represent the mean values of simulated distributions, 1.24 unconfined (blue) and 1.29 confined (red), dotted lines those of the experimental distribution (same as left).

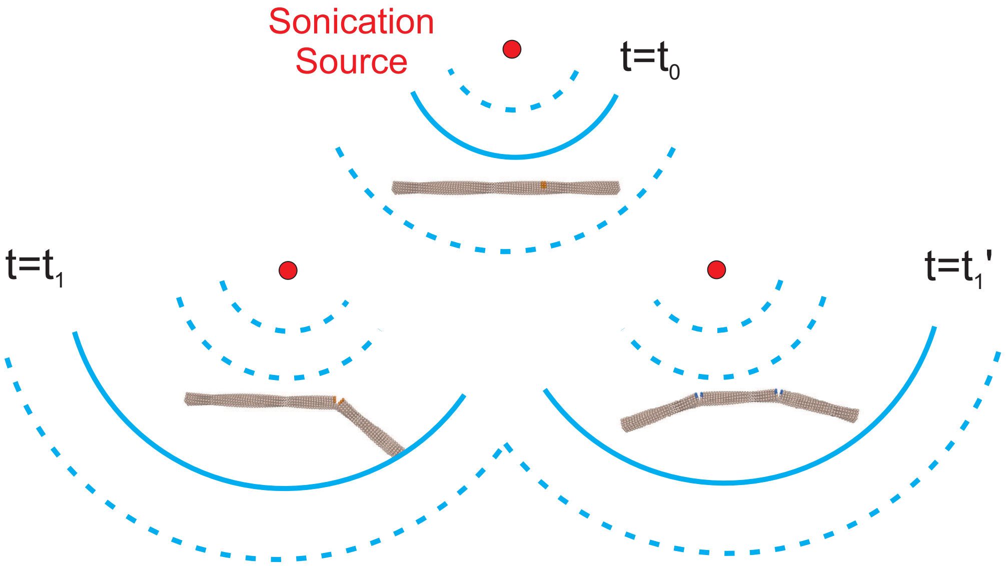

Figure 4: **Simplified schematic of sonication-induced kinks.** Sonication source (red dot) and corresponding wave fronts. Full line represents the same shock wave at different times, $t = t_0$, before hitting the cellulose fibril, t_1 after passing the fibril and breaking in only one point, or $t_1\prime$ after passing the fibril and breaking in 2 points.

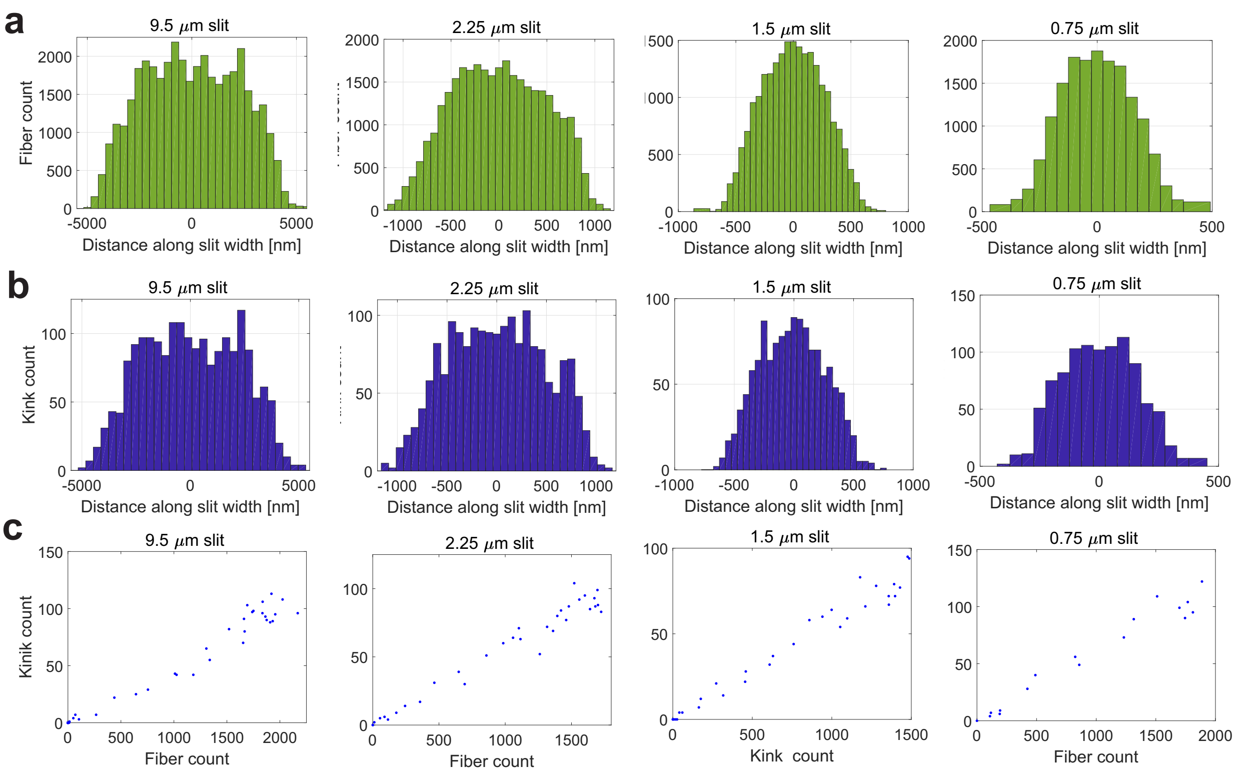

Figure 5: **Fiber and kink distribution within slits** In all rows from left to right increase in confinement **a** Fibril distribution in slits **b** Kink distribution in slits **c** Kink count as a function of Fibril count, where each point represents the kink and fibril count for a identical bins in the distributions in **a** and **b**. The near-linearity of the kink count versus fibril count implies a constant concentration of kinks per fiber throughout the width of the slit.

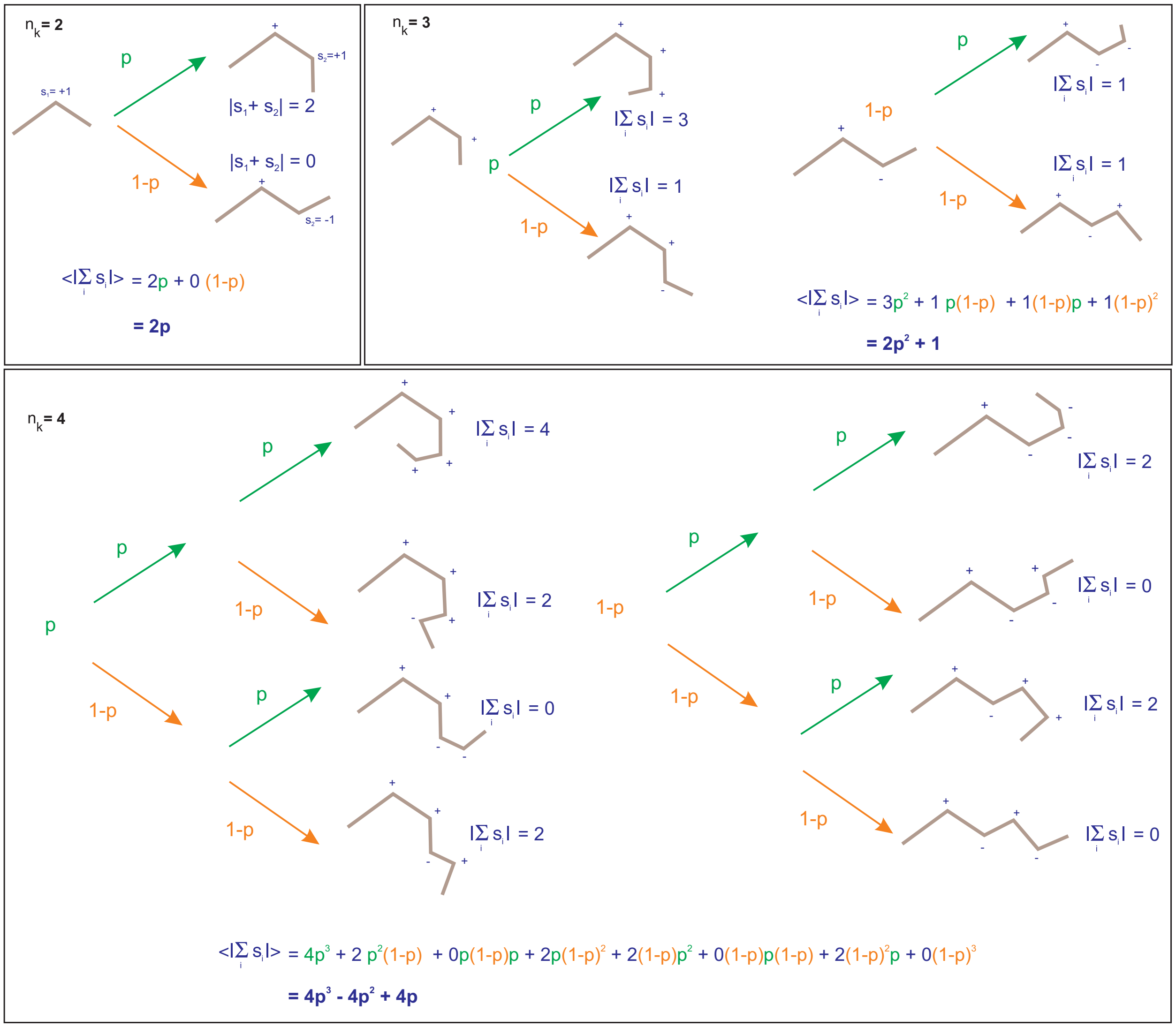

Figure 6: **Probability trees.** Cellulose conformations and associated probability trees for different kink populations assuming a bending probability p of bending in the same direction as the previous kink.
